# Recovery of Terephthalic Acid from Densified Post-consumer Plastic Mix by HTL Process

**DOI:** 10.3390/molecules27207112

**Published:** 2022-10-21

**Authors:** Ilaria Agostini, Benedetta Ciuffi, Riccardo Gallorini, Andrea Maria Rizzo, David Chiaramonti, Luca Rosi

**Affiliations:** 1 Renewable Energy Consortium for R&D (RE-CORD) Viale J. F. Kennedy, 182, 50038 Scarperia e San Piero, Italy; 2 Department of Chemistry “Ugo Schiff”, University of Florence, Via della Lastruccia, 3-13, 50019 Sesto Fiorentino, Italy; 3 “Galileo Ferraris” Energy Department, Polytechnic of Turin, Corso Duca degli Abruzzi 24, 10129 Torino, Italy

**Keywords:** terephthalic acid, hydrothermal liquefaction, PET, plastic waste, chemical recycling, circular economy

## Abstract

In this study, we investigate the hydrothermal liquefaction (HTL) of PET separated from a densified postconsumer plastic mix, with the aim of recovering its monomer. This second raw material is made up of 90% polyolefin, while the remaining 10% is made up of PET, traces of metals, paper, and glass. After preliminary separation by density in water, two batch experiments were performed on the sunken fraction (composed mainly of PET) in a stainless steel autoclave at 345 °C for 30 and 20 min. Both trials resulted in similar yields of the three phases. In particular, the solid yield is around 76% by weight. After a purification step, this phase was analyzed by UV–Vis, ^1^H-NMR, and FTIR spectroscopy and resulted to be constituted by terephthalic acid (TPA), a product of considerable industrial interest. The study proved that the hydrothermal liquefaction process coupled with density separation in water is effective for obtaining TPA from a densified postconsumer plastic mix, which can be used for new PET synthesis.

## 1. Introduction

Terephthalic acid (TPA) is one of the most interesting petrochemicals at an industrial level [1]. In fact, it constitutes the building block for the synthesis of numerous polycondensed polymers, in particular polyethylene terephthalate. In Europe alone, the demand for this polymer reached 4.1 Mt in 2020 [2]. Currently, terephthalic acid is obtained through a process developed by the American Amoco Group, which consists of the oxidation of p-xylene with oxygen. The reaction is carried out in acetic acid, and the catalyst used is cobalt (or manganese) acetate and bromide [3]. With the decrease in oil stocks, there is a growing need to develop alternative routes to obtain chemicals since xylenes are produced by the catalytic reforming of naphtha. From the perspective of a full circular economy, chemicals with high added value can be obtained from plastic waste thanks to chemical recycling. This not only makes it possible to reduce pollution but also to reduce the consumption of virgin oil and protect human health and the environment [4].

Among the various chemical recycling technologies [5], hydrothermal liquefaction (HTL) has aroused great interest in recent years. This is a thermochemical process that takes place in the presence of water in its sub- or supercritical condition. HTL has been extensively studied for biomass conversion to obtain oil, named biocrude, with a high energy content [6]. Different types of biomasses have been subjected to the HTL process, such as lignocellulosic wastes, algae [7], and also a more heterogeneous one made up of a combination of both [8]. The main advantage of the HTL process is that it allows working with wet raw materials, eliminating the need for drying as a pretreatment, necessary instead for the gasification and pyrolysis processes [9]. Furthermore, thanks to its bifunctionality (chemical and thermal properties), this type of process is well suited for the valorization of heterogeneous and biocontaminated wastes, which, due to their nonhomogeneous composition, would be landfilled or incinerated. So, different types of plastic (in terms of chemical nature, color, size, and physical properties) can be processed at the same time and in the same reaction environment [10]. Moreover, the high temperature that characterizes the operating conditions of HTL processes destroys all biological active organisms (such as pathogens, viruses, and bacteria), obtaining safe products [11].

Water near its critical point (Tc = 374.1 °C, Pc = 22.1 MPa) acts at the same time as a reactant and solvent [12], taking part in the depolymerization reactions of plastics. In these conditions, the properties of water change as compared to room temperature, as shown in Table S1 in the Supplementary Materials, allowing controlling the degree of hydrolysis and the solubility of the materials processed [13,14]. Through the HTL process, four distinct phases are always obtained, whose yield and composition depend on the starting feedstock and the conditions applied:

- A liquid/semi-solid organic phase, consisting of organic compounds with low molecular weight, derived from depolymerization reactions;
- An aqueous phase, with dissolved organic compounds called water soluble (WSO);
- A solid phase, formed by no reacted compounds, inorganic, carbonaceous products;
- A gaseous phase, containing the gases produced during feedstock degradation reactions.

The yield of the various fractions changes according to the applied operating conditions: short reaction times favor the yield of oil, while long reaction times increase the yield of char and gas [15].

In the literature, there are already available studies regarding the application of the HTL process for the conversion of plastics.

For example, dos Passos [16] reported a comprehensive overview regarding the ease of conversion, yield, and composition of products obtained from HTL under subcritical conditions (350 °C, 20 min, with and without KOH) of the most used single polymers. According to their study, polymers are broken down by HTL into lower-molecular-weight products thanks to water acting as a nucleophile. The degree of hydrolysis and the type of products that are recovered strictly depend on the chemical structure of the starting polymer; in fact, each plastic has its own different degradation behavior. In the specific case of PET, a high yield (70%) of solid product was obtained. FTIR analysis of the latter showed it to be high-purity terephthalic acid. There are other studies in the literature showing the effectiveness of HTL processes using water under subcritical conditions in the treatment of postconsumer PET. For example, in the study by Darzi et al. [17], PET coming from plastic bottles was subjected to an HTL process, working at a temperature of 300 °C (~10 MPa) to 90 min. In these conditions, over 75% of the PET was recovered as solid-phase TPA.

This study aims to enhance the PET fraction of industrial waste, coming from a postconsumer plastic densification process, by means of hydrothermal liquefaction. First, separation by density in water was carried out, to separate the polyolefin fraction from the one containing PET. Subsequently hydrothermal liquefaction reactions were carried out on the polyester fraction at 345 °C for 20 and 30 min, to recover terephthalic acid as a chemical of high industrial interest.

## 2. Materials and Methods

### 2.1. Feedstock

The raw material used in this study is a production waste coming from the process of collection and densification of postconsumer plastic waste. As stated by the manufacturer, the polyolefin content is greater than 90%. Furthermore, the product complies with the provisions of REGULATION (EC) N. 1907/2006 (REACH), in this regard to restricted substances and Substances of Very High Concern (SVHC), by REGULATION (CE) 2019/1021 (POP), and is not subject to labeling obligations according to REGULATION (EC) N.1272/2008 (CLP). This raw material also complies with the UNI technical standard 10667-16, which makes it usable to be extruded in various shapes and/or to produce products by injection molding. Currently, however, its most common application concerns its direct use as an energy source and reducing agent in electric arc furnaces for steel production [18].

**Figure 1:**
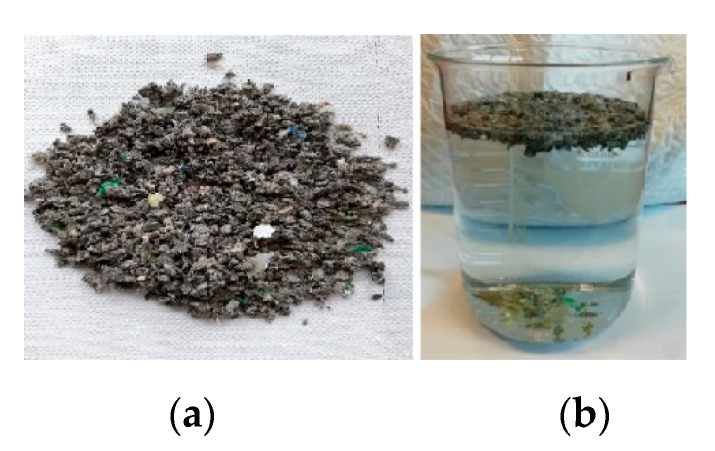
Feedstock before (**a**) and after (**b**) density separation in water.

The waste appears as 3–10 mm solid flakes with different colors (Figure 1a). It is very heterogeneous from a chemical point of view and consists of different polymers, metal, paper, and glass fragments.

The chemical valorization of a heterogeneous material necessarily passes through the separation of components to obtain streams that are as chemically homogeneous as possible. For this purpose, the feedstock was subjected to separation by density in water. This simple method, as shown in Figure 1b, allows separating polyolefin-based polymers with a density below water (about 0.9 g·cm^−3^) from PET with a density higher than water (about 1.3–1.4 g·cm^−3^).

In this way, two distinct fractions were obtained: a floating fraction formed almost entirely of a dark gray material in granular form, and a sunken one formed of various colored flakes and fragments with a metallic and glassy aspect. The two fractions were subsequently dried in an oven at 105 °C until constant weight and characterized.

### 2.2. Experimental Procedures

The hydrothermal liquefaction experiments were carried out in a 160 mL stainless steel Parr autoclave (Parr Instrument Company, Moline, IL, USA). The autoclave features two main valves (input and output) and is equipped with a stirrer, a J-type thermocouple, and a pressure sensor (model Parr 4842, in which pressure is displayed with 1 psi resolution and 10 psi accuracy). The heating system consists of an electric heating mantle rated for 1 kW and regulated by an electronic controller. Two sets of experiments were conducted in duplicate using ultrapure water (0.05 μS·cm^−1^) and varying only the reaction time (respectively, 20 and 30 min). Experiments were conducted in subcritical conditions (345 °C, 150 bar) under stirring at 220 rpm. The temperature used in the process is dictated by instrumental constraints. For each experiment, 3 g of sunken fraction and 50 g of ultrapure water were used, with a percentage ratio by weight of dry feedstock/water of 6%, as suggested by recent literature [16].

The average heating rate for each test was 8 °C·min^−1^ (Figure S1, Supplementary Materials). The reaction time was measured only after reaching the target temperature. The time needed to reach 345 °C was 41 min. As described by Darzi et al. [17], it should be noted that the melting temperature of PET is exceeded before the process temperature is reached and the depolymerization reactions can already begin.

At the end of the reaction, the autoclave was quenched by immersion in a cold-water bath and then weighted. The gas produced was vented and the reactor reweighted to determine the mass of gas produced during the reaction. Reaction products were collected using a procedure that consists of different steps. Firstly, the content inside the reactor was washed with dichloromethane (DCM) and filtered under vacuum over a glass microfiber filter (1 μm). The solid recovered on the filter was dried in an oven at 105 °C until constant weight. The liquid phase was transferred to a 15 mL centrifuge tube and centrifuged for 5 min at 4000 rpm. Two distinct liquid phases (DCM and water) were collected separately using a separating funnel. The organic fraction containing DCM was subjected to evaporation under reduced pressure to obtain the oil. The solid fraction was subsequently purified following three phases: first, solubilization of the solid product in a 1 M NaOH solution was performed to obtain sodium terephthalate, followed by vacuum filtration through a Buchner funnel to remove the undissolved impurities, and finally precipitation of the terephthalic acid by adding sulfuric acid. The precipitate was then filtered, washed with water, and dried to constant weight.

FTIR spectra of the samples were obtained using a Shimadzu IR Tracer-100 spectrometer, equipped with QATR™ 10 Single-Reflection ATR with a Diamond Crystal (Shimadzu, Nishinokyo Kuwabara-cho, Kyoto, JP), operating with a maximum resolution of 0.25 cm^−1^ and a spectral range in the mid-IR region (4100–500 cm^−1^). The spectra were acquired in transmittance (%) using 45 scans. Measurements were carried out in triplicate for each sample.

The content of carbon, hydrogen, and nitrogen (CHN) of the samples was determined using a Thermo Scientific Flash Smart Elemental Analyzer CHNS/O (Thermo Fisher Scientific, Waltham, MA, USA), according to UNI EN ISO 16948 (UNE EN ISO 16948, 2015). Measurements were carried out in triplicate, and the average value was reported. The oxygen content was calculated by the difference:

(1) $$O\;\left(\%\right)=100-\left(\%C+\%H+\%N\right)\times 100$$

DSC experiments of feedstock were performed using DSC 25 (TA Instruments New Castle, DE, USA). The sample and reference furnaces were purged with nitrogen gas at a flow rate of 40 mL/min. Calibration of the sensor temperature and of the heat-flow rate was performed according to standard procedures, using indium as a calibrant. Samples with a mass of about 4 mg were placed in aluminum pans (Perkin-Elmer, Waltham, MA, USA). The samples were heated from 40 °C to 300 °C at a rate of 10 °C/min, followed by isothermal equilibration at 300 °C for 1 min to eliminate any thermal history. Then, they were cooled at a rate of 5 °C to room temperature. Two other scans were conducted in the same way to ensure the reproducibility of the measurement.

The concentration of inorganics was determined in an Agilent MP4200 ICP-OES (Agilent, Santa Clara, CA, USA). Before the analysis, samples were mineralized with 3 mL of hydrogen peroxide and 5 mL of nitric acid in a Milestone Start D microwave digestion system (Milestone, Sorisole (BG), IT) to obtain clear acidic solutions. Measurements were carried out in triplicate for each sample and the average was reported.

The higher heating value (HHV) of the samples was determined using a Leco AC500 (Leco, St. Joseph, MI, USA) apparatus according to UNI EN ISO 18125. A 0.3 g amount of material was used for each test, and since the samples were very heterogeneous, measurements were carried out in triplicate and the average value was reported. ^1^H-NMR spectra were recorded on a Varian Gemini 200 MHz (Varian, Palo Alto, CA, USA). The ^1^H-NMR spectroscopic data are reported as (multiplicity, integration). The following abbreviations were used to designate multiplicities: s = singlet; bs = broad singlet. Chemical shifts were determined relative to the residual solvent peak (DMSO: 2.50 ppm for ^1^H-NMR). 

UV absorption measurements were carried out using the Cary 4000 UV-Vis Spectrophotometer (Agilent, Santa Clara, CA, USA). Measurements were performed on TPA standard and solid product according to Yang et al. [19].

Qualitative and semi-quantitative analyses of the organic compounds in the oil were performed by GC 2010 with the Shimadzu GCMS-QP2010 mass spectrometer (Shimadzu, Nishinokyo Kuwabara-cho, Kyoto, Japan) equipped with the Zebron ZB-5HT INFERNO (Phenomenex, Torrance, CA, USA) column (length, 30 m; internal diameter, 0.250 mm; film diameter, 0.25 μm). A 40 μg amount of oil was solubilized in 3 mL of acetone, and then 1 mL of the solution was injected into the GC-MS apparatus. The analysis was performed with a He column flow of 2.02 mL·min^−1^ with an initial temperature of 40 °C (holding time, 10 min), increased to 200 °C (heating rate, 8 °C min^−1^; holding time, 10 min), and then to 280 °C (heating rate, 10 °C min^−1^; holding time, 30 min). The mass spectra acquired were compared with those contained in the NIST17 library.

### 2.3. Determination of the Yields

From each HTL experiment, four different fractions were obtained: solid, gas, oil, and aqueous fraction. The weight yields of each fraction were calculated using Equations (2)–(5) below, in which W indicates the weight:

(2) $$\mathrm{Solid}\;\mathrm{residue}\;\mathrm{yield}\;\left(\%\right)=\frac{Wsolid}{Wfeedstock}\times 100$$

(3) $$\mathrm{Oil}\;\mathrm{product}\;\mathrm{yield}=\frac{Woil}{Wfeedstock}\times 100$$

The weights of the solid and oil fractions were calculated with the balance, while the amount of gas produced during the reactions was determined as the weight difference of the reactor weight before the heat-up and after venting the gaseous products at the end of the cooling process.

(4) $$\;\mathrm{Gas}\;\mathrm{yield}\;\left(\%\right)=\frac{Reactor\;weight\;after\;venting-initial\;reactor}{Wfeedstock}\times 100$$

Water-soluble organics and unrecovered products (i.e., all losses due to experimental operations) were estimated by difference once the yields of solid, gas, and oil were known:

(5) $$\;\mathrm{WSO}+\mathrm{unrecovered}\;\left(\%\right)=100-\left(solid\;residue\;yield+oil\;yield+gas\;yield\right)\times 100$$

## 3. Results and Discussion

### 3.1. Feedstock Characterization

The feedstock was dried at 45 °C for 3 days, and then 20 g was subjected to density separation in water. Due to the high heterogeneity, this process was repeated three times. The floating fraction constitutes 90 ± 1.5% by weight of the feedstock, while the sunken fraction constitutes 10 ± 1.5%. In the higher-density fraction, in addition to polymers, there are also contaminants such as glass, metal, and paper. This fraction consists of 91.6 ± 1% by weight of plastic waste, 2.4 ± 1% of metal, 4.6 ± 1% of glass, and 1.4 ± 1% of paper. The metal component does not consist of iron residues but only aluminum.

**Figure 2:**
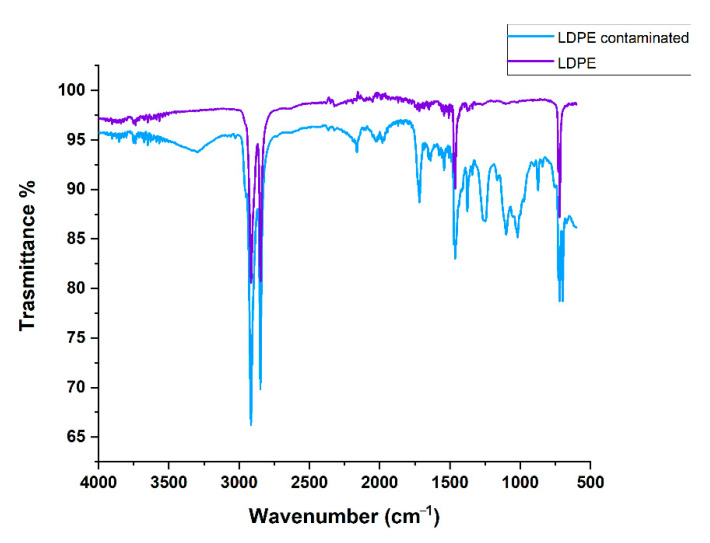
IR spectra of the floating fraction of the feedstock: LDPE (violet line) and polyethylene contaminated with aromatic polyester (light blue line).

The IR spectra acquired on the floating fraction are reported in Figure 2. The chart shows the typical adsorptions expected for pure low-density polyethylene (purple line) but also for polyethylene contaminated with aromatic polymers containing oxygen such as PET (blue line). In fact, in addition to peaks of polyolefin polymers (2916 cm^−1^, 2848 cm^−1^: -C-H stretching; 1471 cm^−1^: -C-H bending; 720 cm^−1^, 698 cm^−1^: -C-H rocking), peaks associated with polyester are visible also (3026 cm^−1^: =C-H stretching; 1716 cm^−1^: -C=O stretching; 1637 cm^−1^, 1541 cm^−1^: -C=C stretching; 1247 cm^−1^, 1101 cm^−1^: -C-O stretching; 873 cm^−1^ p-substituted aromatic ring rocking). In the densification process, some PET fragments are in fact incorporated into polyolefin fragments.

**Figure 3:**
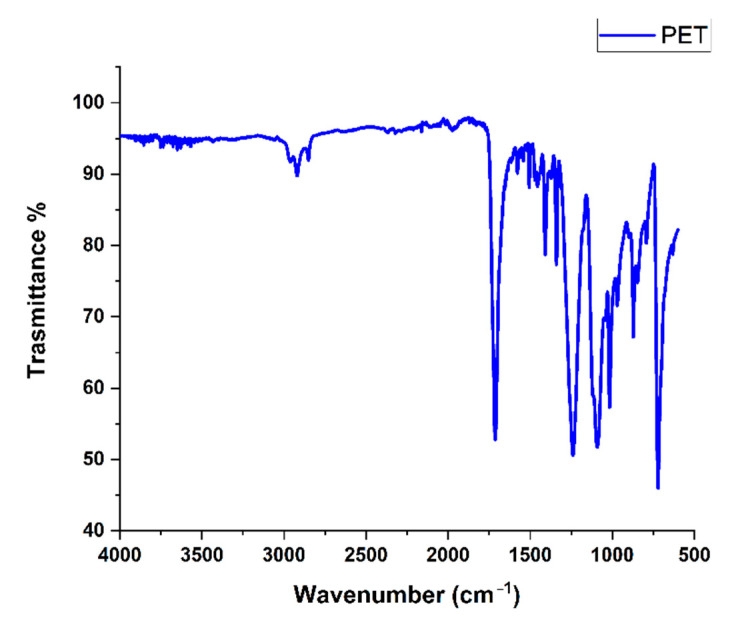
IR spectrum of PET presents in the sunken fraction of the feedstock.

The sunken fraction was more heterogeneous in appearance, and it consisted of different-color polymer flakes, from whose spectra it was possible to identify the characteristic adsorptions of polyesters and in particular of PET (Figure 3) [20].

The DSC analysis of the fragments confirmed the hypothesized composition by FTIR; in fact, the values of Tg, Tc, and Tm are those expected for PET [21]. The thermograms of some fragments are shown in Figure S2 in the Supplementary Materials.

The concentration of inorganics in the feedstock is reported in Table S2 (Supplementary Materials). The ICP-OES analysis highlights the presence of some elements in high quantities, such as Al, Ca, Mg, Na, Si, and Ti. Their presence is linked to the use of additives, as described in detail in the literature [22].

The elemental composition on the dry basis of the feedstock and the two fractions obtained through the separation in water is given in Table 1.

**Figure 4:**
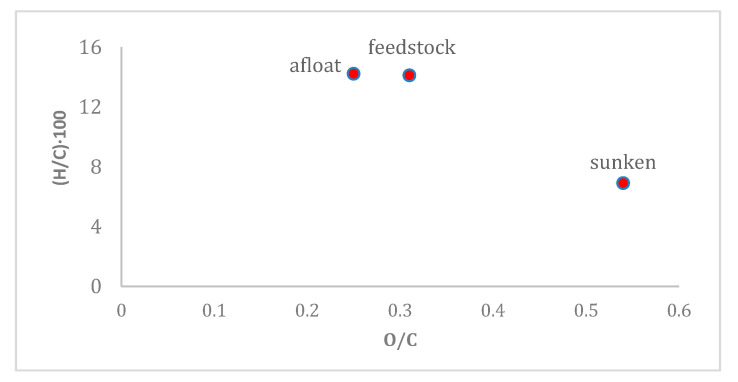
Van Krevelen diagram of the two fractions (afloat and sunken) and of the initial feedstock.

The floating fraction has a higher percentage of carbon than the sunken one which appears richer in oxygen. The H/C and O/C ratios of both the feedstock and the two fractions are shown in the following van Krevelen diagram (Figure 4). The obtained values suggest that the fraction having lower density is better suited to produce fuels, thanks to its high H/C ratio and low O/C ratio, which will have a higher calorific value compared to the sunken part.

The calorific values (HHV) of the initial feedstock and of the two fractions obtained from the separation in water are reported in Table 2.

The calorific value determines the energy content of the material. As already observed, the afloat fraction has a calorific value higher than the sunken fraction and the initial feedstock. Its value is, however, lower than the expected typical figure for pure polyolefin polymers (43–46 MJ Kg^−1^). This can be explained by considering the contamination of the polyolefin fraction with the polyester fraction, which inevitably occurs during the process.

### 3.2. HTL Product Yields

Two HTL processes with different residence times were performed on the sunken fraction. In this way, the chemical recycling process of mixed wet wastes, following a density pretreatment, was reproduced on a laboratory scale. On the other hand, the lower density fraction, rich in polyolefin, is not suitable for being subjected to this type of process due to the absence of reactive sites in the polymer chains and because the working temperature (345 °C) is significantly lower than the thermal cracking temperature of polyolefin (around 450 °C). Thus, in the process under consideration, the polymer chains would remain undecomposed. This fraction thanks to the high H/C ratio is suitable for producing liquid fuels.

**Figure 5:**
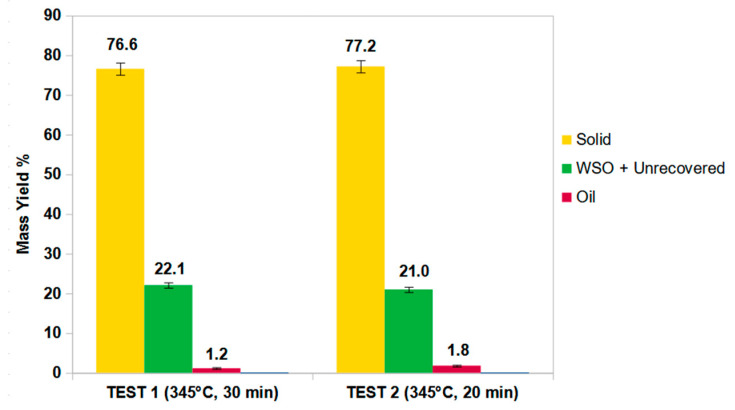
Yields with relative error from the two tests at 345 °C with different residence time.

Since the sunken fraction is formed mainly by PET, for the HTL tests, the same operating conditions were chosen that were already used for pure PET feedstocks [16]. For the first test, a residence time of 30 min was chosen and for the second one 20 min. The purposes were to diminish the residence time to promote energy recovery as much as possible, to reduce the related costs, and to apply the same operating conditions in use in modern HTL biomass plants to the treatment of plastic wastes. In general, the current subcritical HTL technology is based on rapid heating speed reactors, which work close to 300–360 °C and with a moderate residence time (15–20 min) [11]. The yields obtained from both tests are shown in Figure 5.

As can be seen, a 10 min reduction in the reaction time does not lead to significant differences in product yields, resulting in significant energy savings. In both processes, the solid fraction is the majority product with a yield of over 76%. Considering the heterogeneity of the sample and therefore the variable quantity of PET for each test, the yields of the two processes are comparable. Gas formation was not revealed during the experiments. This agrees with the data in the literature in which noncatalyzed processes always generate small quantities of gas [16].

### 3.3. HTL Products Characterization

#### 3.3.1. Solid Characterization

The solids obtained from both tests consist of a brown dust in which lamellar crystals are visible.

**Figure 6:**
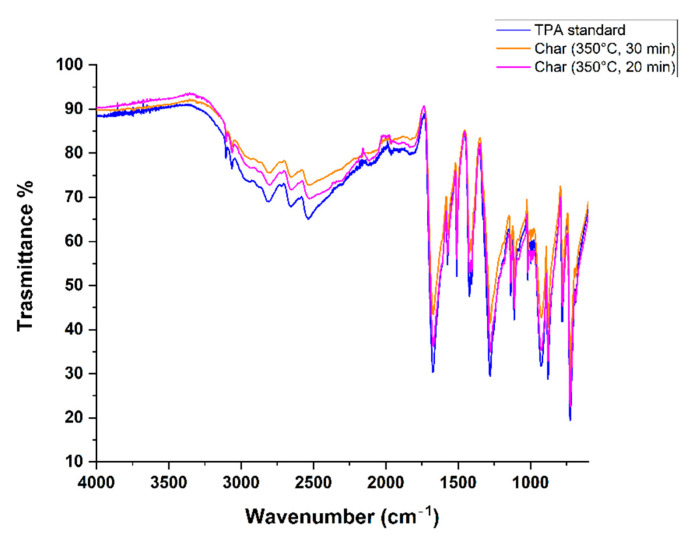
IR spectra of the solids obtained from test 1 (orange; 345 °C, 30 min), from test 2 (magenta; 345 °C, 20 min), and TPA standard (blue).

The FTIR spectra of the obtained samples were compared with those of pure terephthalic acid (TPA standard). As can be observed in Figure 6, there is a complete superposition of the spectra, which confirms the achievement of terephthalic acid, as described by the literature [16,23,24,25]. O-H stretching of carboxylic acid is clearly visible at 3160–2150 cm^−1^, and =C-H stretching at 3101 cm^−1^ and 3064 cm^−1^, C=O stretching at 1670 cm^−1^, C=C stretching of the aromatic ring at 1571 cm^−1^ and 1508 cm^−1^, and C-O stretching at 1276 cm^−1^ and 1101 cm^−1^ are also visible and typical of aromatic carboxylic acid compounds.

An elementary analysis was also carried out on the two solids, whose results are shown in Table 3.

The weight percentages of C, H, and N are in good agreement with those obtained for the solid phase of the HTL process of pure PET, as described in the literature [16].

The empirical formula of both solids was calculated by dividing the wt. % of each element by its atomic weight, obtaining for both tests the minimum formula: C_4_H_3_O_2_. The molecular formula of terephthalic acid C_8_H_6_O_4_ was obtained, doubling the minimum one, considering its molecular weight (166.13 g∙mol^−1^). The number of unsaturations was calculated according to Equation (6) and is equal to six: three double bonds, one aromatic ring, and two C=O double bonds.

(6) $$\;\mathrm{Degree}\;\mathrm{of}\;\mathrm{unsaturation}=nC-\frac{nH}{2}-\frac{nX}{2}+\frac{nN}{2}+1=8-3+1=6$$

X = F, Cl, Br, I.

The results are once again in agreement with the terephthalic acid molecule.

**Figure 7:**
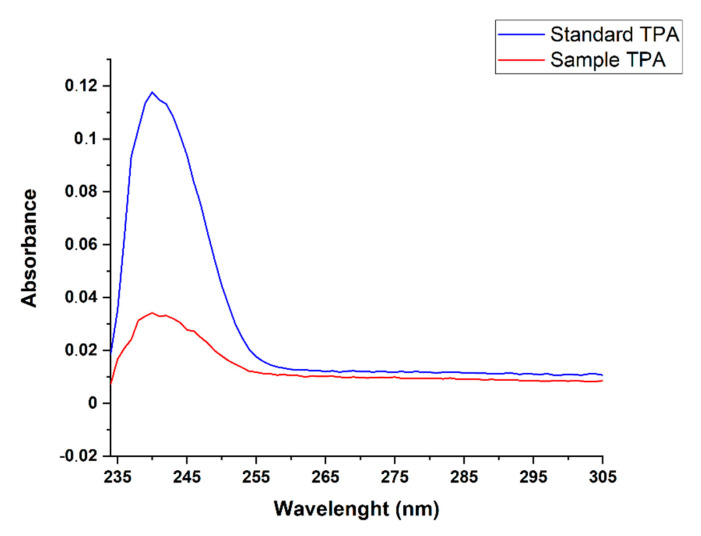
Comparison between the UV–Vis spectrum of the product obtained from test 2 (20 min) with that of the standard TPA.

Spectroscopic analyses were carried out on the purified product to confirm its nature. Figure 7 depicts a comparison between the UV–Vis spectra of standard TPA (blue line) and our purified sample (red line). Our sample exhibits the characteristic absorption at 240 nm corresponding to the electronic transitions π→π* of conjugated C=C sp2 domains of aromatic ring [26].

An NMR spectrum was also recorded for a more detailed characterization of the solid (Figure S3 in the Supplementary Materials). It shows a singlet at 8.10 ppm due to the presence of the aromatic ring and broad singlet at 13.20 ppm due to the presence of the two mobile protons of the carboxyl group [27].

#### 3.3.2. Oil Characterization

The oil obtained through HTL consists of a viscous liquid of brown/black color. The IR spectra of the oils are reported in Figure S4 in the Supplementary Materials. In both oil phases, the typical adsorptions of aromatic compounds (3059 cm^−1^ and 3022 cm^−1^: stretching =C-H; 1600 cm^−1^ and 1570 cm^−1^: stretching C=C; 710 cm^−1^ and 696 cm^−1^: rocking =C-H of the monosubstituted aromatic ring; 3500–3000 cm^−1^: stretching O-H, carboxylic acids/esters, and alcohols; 1680 cm^−1^: stretching C=O, 1260 cm^−1^: stretching C-O) are visible.

**Figure 8:**
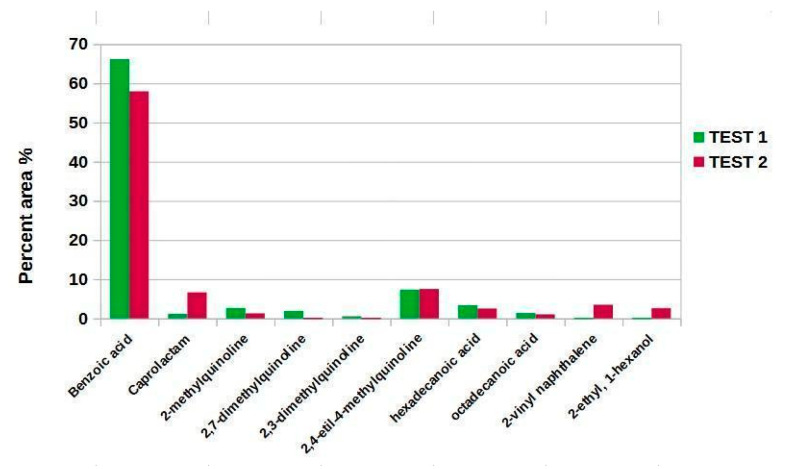
Comparison between the relative percentage areas of compounds present in the oils of Tests 1 and 2.

The composition of the two oil fractions was determined by GC-MS analysis. Figure 8 compares the relative percentage areas of the compounds identified in the oils of the two tests.

Benzoic acid derives from the degradation through decarboxylation of terephthalic acid, which in turn derives from the depolymerization of PET. According to the literature above 300 °C, terephthalic acid is converted to the corresponding monoacid through decarboxylation reaction. This decomposition pathway increases the yield of benzoic acid and gas (CO_2_) and decreases that of monomer [28].

Caprolactam can also derive from bis(1-caprolactam) carbonyl (CBC), used as a chain extender in the synthesis of PET. Chain extenders serve to modulate the molecular weight and modify the rheology of the polymer in the melt state (e.g., viscosity). The chain extenders have at least two functional groups capable of giving additional reactions with the hydroxyl or carboxyl groups of the polyester. CBC has a melting point of 105 °C and during PET processing it can react with the terminal hydroxyl functional groups present on the chains [29]. The viscosity can be modulated based on the amount of CBC added to the polymer. Usually, this additive is present for 5 wt. %.

The 2- and 4-methyl derivatives of quinoline are used as precursors in the production of cyanine dyes. These dyes are mainly green or blue in color and have the general formula R_2_N[CH=CH]_n_CH=N+R_2_ 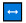 R_2_N+=CH[CH=CH]_n_NR_2_, in which nitrogen is part of a heterocycle [30]. It is possible to identify two centers that contain a nitrogen atom and an alternation of single and double bonds between them. The dyes used in the production of polymers can be present up to 10% by weight [31].

Long-chain fatty acids can also be derived from the decomposition of nucleating agents used in PET synthesis to promote the crystallization of semi-crystalline polymers. One of the major limitations of PET is its slow crystallization from the melt state, which takes a long time to take place. In these conditions, large crystals are formed, which give opacity and fragility to the polymer. Nucleating agents lead to several small spherulites, rather than a few large ones, giving enhanced properties such as flexural modulus and heat deflection temperature. These additives are typically alkali metal salts of high molecular weight [32], but waxes consisting of mixtures of saturated carboxylic acids with a linear chain (26–34 carbon atoms) are also used (e.g., montanic wax). They have low volatility and high thermal stability [24]. Seshasayee et al. [33] identified hexadecanoic and octadecanoic acid from the HTL process of 30 wt. %-reinforced PET pellets with glass particles, confirming the presence of these compounds as additives.

Other compounds were also identified in addition to those presented in Figure 8, but their small relative area (less than 0.5%) meant it was not possible to correctly identify them, and thus they were omitted.

## 4. Conclusions

In this work, terephthalic acid was recovered by hydrothermal liquefaction in subcritical conditions of real industrial waste coming from a postconsumer plastic densification process.

Through a first separation by density in water, it was possible to divide the initial plastic mix into two fractions: one representing 90% formed by polyolefin polymers (floating), and one representing 10%, containing mainly PET (sunken). Two hydrothermal liquefaction processes were then carried out using water under subcritical conditions (345 °C and 150 bar) on the higher-density fraction, varying only the reaction time (30 and 20 min). Thanks to the action of water as a nucleophile in the hydrolysis reactions of PET, it was possible to obtain a high yield of terephthalic acid in the solid fraction (over 76%). This product with high added value can be used to carry out new PET synthesis. Unlike other methods examined in the literature [34], HTL allows the conversion of PET into terephthalic acid with high yields by working at lower temperatures and in the absence of a catalyst, which would contaminate the solid phase. Furthermore, compared to other HTL studies [17], the process conditions were optimized, obtaining the same yield of terephthalic acid but carrying out the reactions for shorter times. The time required to complete the process drops from 90 to 20 min when increasing the temperature by 45 °C and the pressure by 50 bar. The high yield in terephthalic acid is promising for making economic assessments relating to the sustainability of the process. The obtained result appears to be in excellent agreement with the circular economy approach of modern sustainable economies. To conclude, thanks to the separation by density in water, it was possible to obtain a fraction formed by polyolefin with a calorific value higher than the starting material, which can be enhanced to obtain fuels.

## Figures and Tables

**Table 1:** Elemental analysis on dry basis of the feedstock and of the two fractions obtained by density separation (afloat and sunken).

	Element	Amount (wt. %)
Feedstock		
	C	68.7
	H	9.7
	N	0.2
	O	21.4
Afloat fraction		
	C	71.6
	H	10.2
	N	0.4
	O	17.8
Sunken fraction		
	C	62.1
	H	4.3
	N	0.0
	O	33.6

**Table 2:** Higher heating value (HHV) of the feedstock and of the two fractions obtained by density separation (afloat and sunken).

Sample	Value (MJ Kg^−1^)
Feedstock	29.1
Afloat fraction	33.9
Sunken fraction	22.9

**Table 3:** Elemental analysis of the solid residues obtained from HTL test 1 (345 °C, 30 min) and test 2 (345 °C, 20 min).

Element	Solid Test 1—Amount (wt. %)	Solid Test 2—Amount (wt. %)
C	57.4	57.4
H	3.7	3.7
N	0.03	0.04
O	38.9	38.8

## Data Availability

The data presented in this study are available on request from the corresponding author.

## Supplementary Materials

The following supporting information can be downloaded at: https://www.mdpi.com/article/10.3390/molecules27207112/s1, Table S1: Physical-chemical properties of water in different conditions; Figure S1: Temperature profile of the HTL reaction; Figure S2: Thermograms of some fragments of the sunken fraction; Table S2: Inorganic content of the feedstock (bdl = below detection limit).; Figure S3: NMR spectrum of the solid product after purification in DMSO-d6; Figure S4: FTIR spectra of the oil phases obtained from the HTL processes: oil from test 1 (blue line; 345 °C, 30 min) and oil from test 2 (orange line; 345 °C, 20 min).

## Sample Availability

Samples of the compounds are available from the authors.
