# Supplementary material for: Recovery of Terephthalic Acid from Densified Post-consumer Plastic Mix by HTL Process
Source: Molecules. 2022 Oct 21;27(20):7112. doi: 10.3390/molecules27207112 (PMC9609039; doi:10.3390/molecules27207112)
Supplement: Supplementary file 1 [file molecules-27-07112-s001.zip › molecules-1973760-supplementary.pdf]

Supplementary Materials

Table S1. Physical-chemical properties of water in different conditions [14].

	Normal water	Subcritical water		Supercritical water	
Temperature (°C)	25	250	350	400	400
Pressure (MPa)	0.1	5	25	25	50
Density (g cm^{-3})	1	0.8	0.6	0.17	0.58
Dielectric constant	78.5	27.1	14.07	5.9	10.5
Ionic product pK_w	14	11.2	12	19.4	11.9
Heat capacity (KJ Kg^{-1} K^{-1})	4.22	4.86	10.1	13	6.8
Dynamic viscosity (mPa s)	0.89	0.11	0.064	0.03	0.07

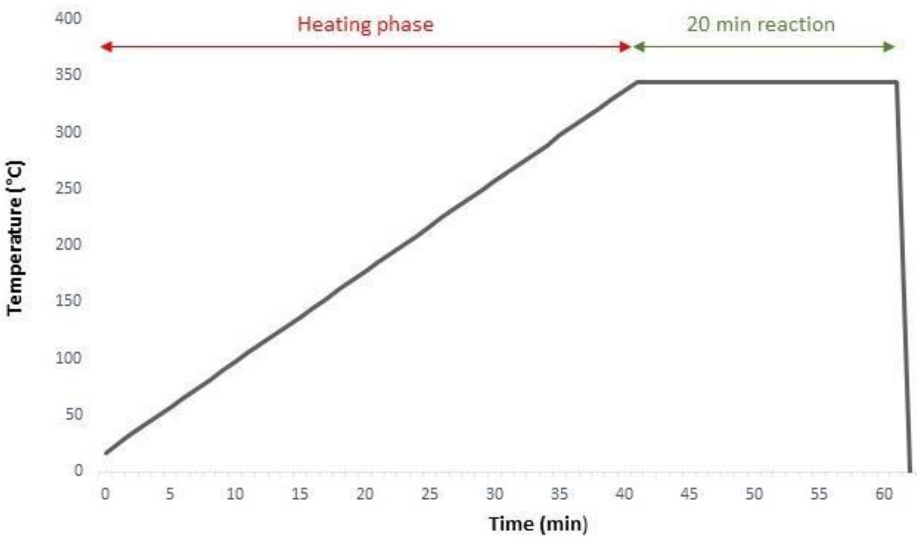

Figure S1. Temperature profile of the HTL reaction

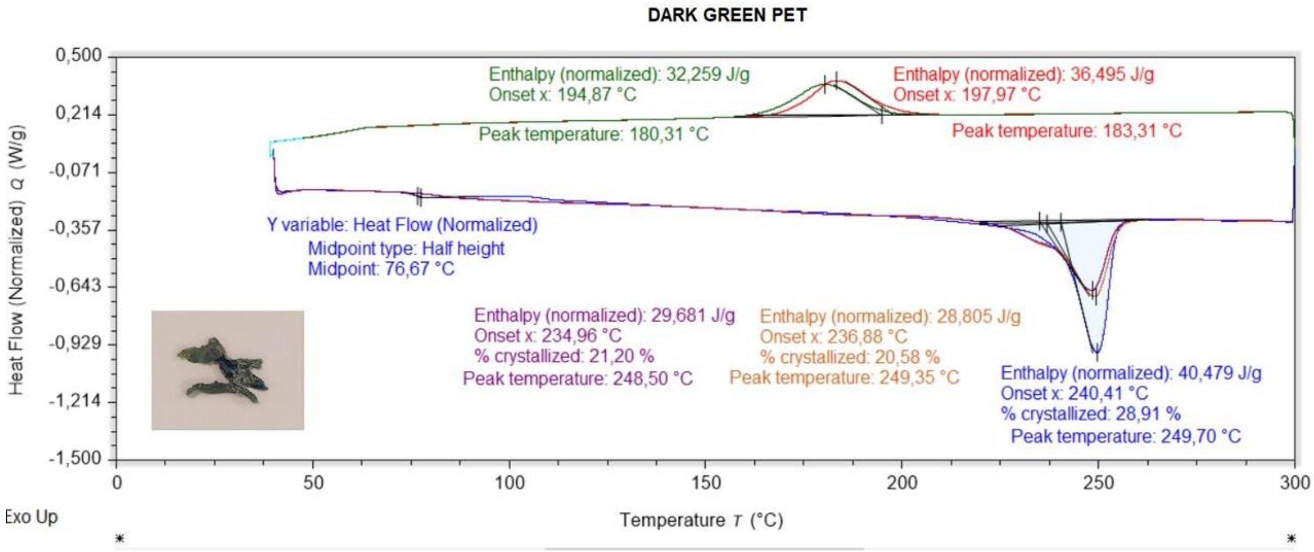

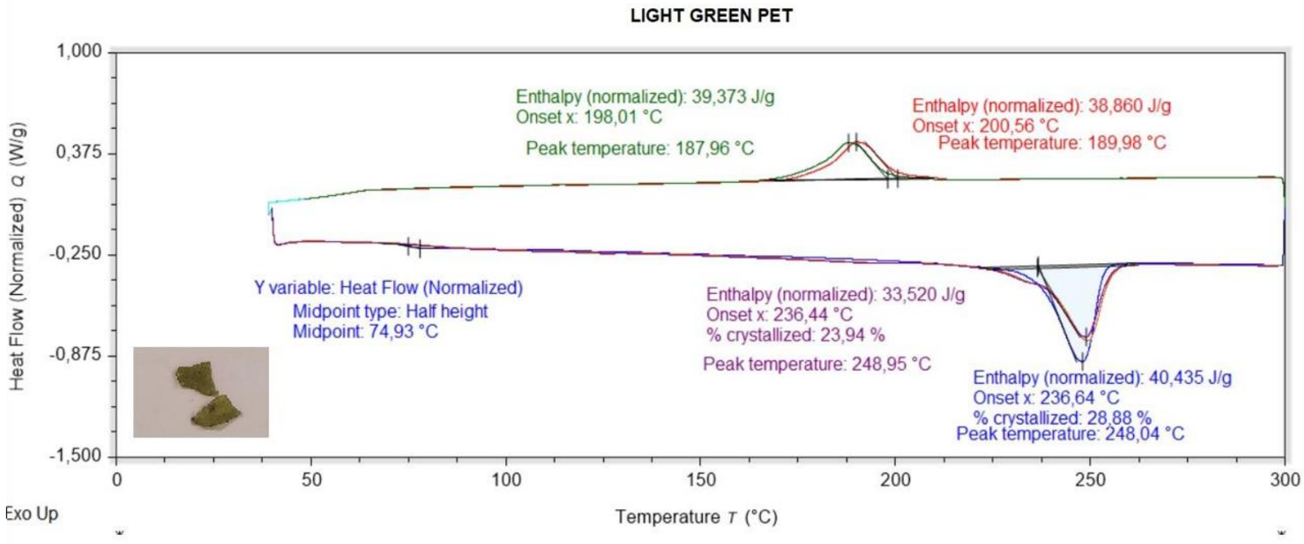

Figure S2. Thermograms of some fragments of the sunken fraction

Table S2. Inorganic content of the feedstock (bdl=below detection limit).

Element	Amount /(mg Kg^{-1})	Method
Hg	<100	ASG 2330 Hg DMA
Al	1816	UNI EN ISO 16968
Ba	8	UNI EN ISO 16968
Ca	4206	UNI EN ISO 16968
Cd	5	UNI EN ISO 16968
Cr	7	UNI EN ISO 16968
Cu	27	UNI EN ISO 16968
Fe	726	UNI EN ISO 16968
K	526	UNI EN ISO 16968
Mg	1405	UNI EN ISO 16968
Mn	18	UNI EN ISO 16968
Mo	2	UNI EN ISO 16968
Na	1447	UNI EN ISO 16968
Pb	9	UNI EN ISO 16968
Si	1400	DIN EN ISO 11885:2009
Ti	1857	UNI EN ISO 16968
Zn	119	UNI EN ISO 16968
Co, L, P, Ni, V	bdl	UNI EN ISO 16968

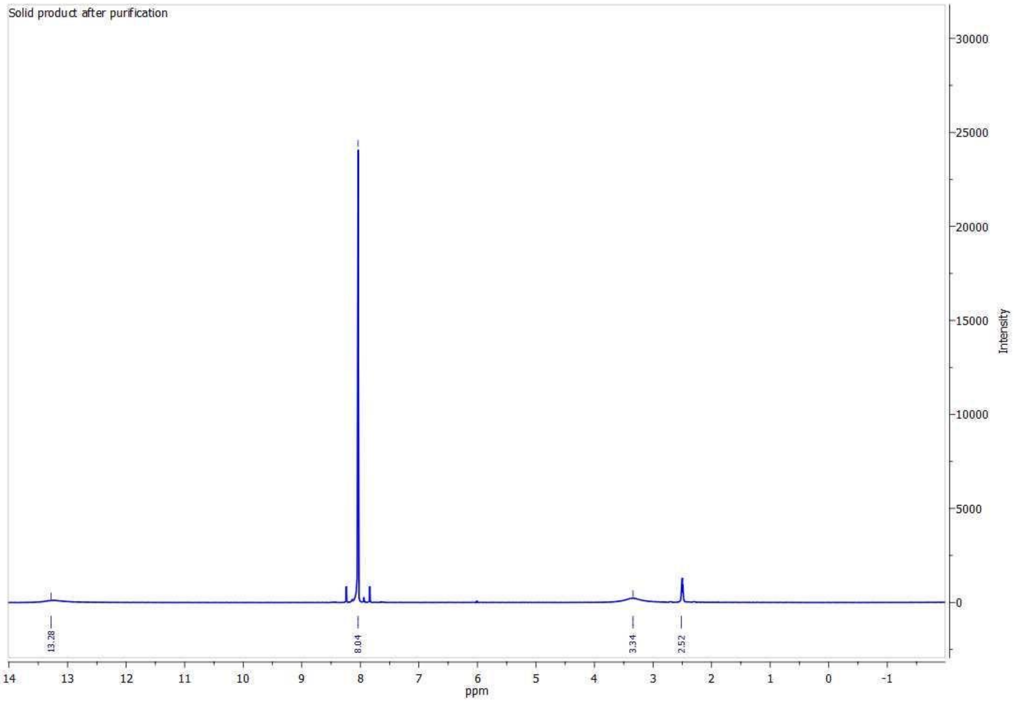

Figure S3. NMR spectrum of the solid product after purification in DMSO-d6

^{1}H-NMR (200 MHz, DMSO-d6): δ= 13.28 (bs, 2H), 8.04 (s, 4H).

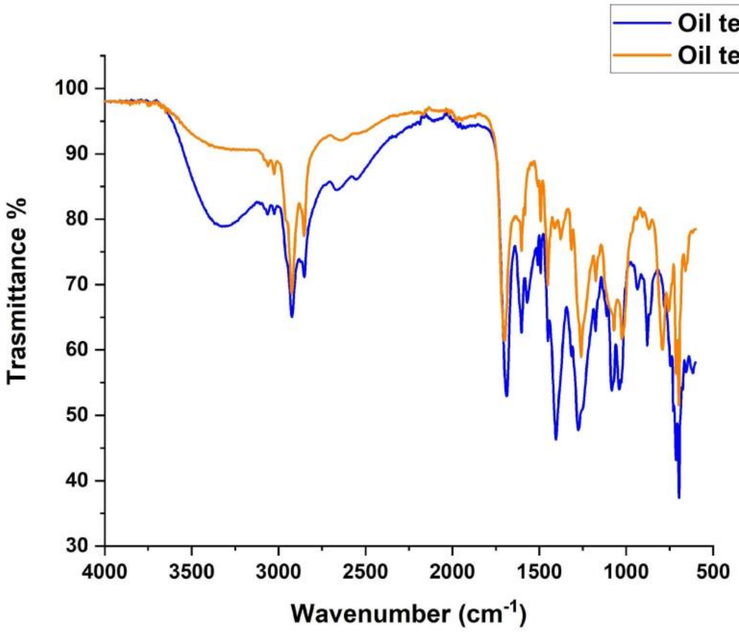

Figure S4. FTIR spectra of the oil phases obtained from the HTL processes: oil from test 1 (blue line; 345°C, 30 min) and oil from test 2 (orange line; 345°C, 20 min).
